# Knowledge of a cancer diagnosis is a protective factor for the survival of patients with breast cancer: a retrospective cohort study

**DOI:** 10.1186/s12885-021-08512-1

**Published:** 2021-06-27

**Authors:** Chen He, Wen Xi Zhu, Yunxiang Tang, Yonghai Bai, Zheng Luo, Jinfang Xu, Hao Wang, Shuyu Xu, Jingzhou Xu, Lei Xiao, Ruike Zhang, Yajing Wang, Jing Du, Yujia Huang, Xiaopan Li, Tong Su

**Affiliations:** 1 Department of Medical Psychology, College of Psychology, Naval Medical University, 800 Xiangyin Rd, Shanghai, 200433 China; 2 School of Nursing and Health Management, Shanghai University of Medicine & Health Sciences, Shanghai, China; 3 Department of Medical Psychology, Changzheng Hospital Affiliated to Naval Medical University, Shanghai, China; 4 Zhoupu Hospital affiliated to Shanghai University of Medicine & Health Sciences, Shanghai, China; 5 Department of Health Statistics, Naval Medical University, Shanghai, China; 6 Department of Cancer Prevention and Vital Statistics, Center for Disease Control and Prevention, Pudong New Area, Shanghai, China; 7 Pudong Institute of Preventive Medicine, Fudan University, Shanghai, China

**Keywords:** Breast cancer, Diagnosis disclosure, Prognosis, Follow-up, Survival, Retrospective cohort study

## Abstract

**Background:**

The health burden of breast cancer is rising in China. The effect of informed diagnosis on long-term survival is not fully understood. This retrospective cohort study aims to explore the association between early informed diagnosis and survival time in breast cancer patients.

**Methods:**

A total of 12,327 breast cancer patients were enrolled between October 2002 and December 2016. Potential factors, including knowing the cancer diagnosis status, sex, age, clinical stage, surgery history, grade of reporting hospital and diagnostic year were, analyzed. We followed up all participants every 6 months until June 2017. Propensity score matching (PSM) was used to balance the clinicopathologic characteristics between patients who knew their diagnosis and those who did not.

**Results:**

By June 2017, 18.04% of the participants died of breast cancer. Before PSM, both the 3-year and 5-year survival rates of patients who knew their cancer diagnosis were longer (*P* < 0.001). After PSM, the above conclusion was still established. By stratified analysis, except for the subgroups of male patients and stage III patients, patients who knew their diagnosis showed a better prognosis in all the other subgroups (*P* < 0.05). Cox regression analysis showed that knowing a cancer diagnosis was an independent risk factor for survival in breast cancer patients (*P* < 0.001).

**Conclusions:**

Being aware of their cancer diagnosis plays a protective role in extending the survival time of breast cancer patients, which suggests that medical staff and patients’ families should disclose the cancer diagnosis to patients in a timely manner. Further prospective studies need to be made to validate our findings.

**Supplementary Information:**

The online version contains supplementary material available at 10.1186/s12885-021-08512-1.

## Background

Breast cancer, lung cancer, and colon cancer are the three most common cancers worldwide. One in eight to ten women will develop breast cancer during their lifetime [1]. Based on the National Cancer Institute (NCI)‘s Surveillance, Epidemiology, and End Results (SEER) program of the United States, the incidence of breast cancer among women aged 20 to 39 years increased from 24.6/1000,000 to 31.7/100,000 from 1975 to 2015. The 5-year survival increased from 74.0 to 88.5% [2]. However, breast cancer continues to be the most common cause of female death in developing countries and second to lung cancer in developed countries. Especially in South America, Africa, and Asia, the incidence of breast cancer is increasing instead of decreasing [3].

Since the 1990s, the incidence of breast cancer in China has increased more than twice as fast as the global rates, particularly in urban areas [4]. By 2008, China accounted for 12.2% of global cases of invasive breast cancer and 9.6% of the related deaths [5]. It is anticipated that the cases of breast cancer in China will increase from less than 60 cases per 100,000 women aged 55–69 years to more than 100 cases per 100,000 women, reaching 2.5 million cases overall by 2021 [6].

The patterns of breast cancer risk in Chinese women are partly aligned with known risk factors for women in developed countries [7]. Reproductive and hormonal factors such as nulliparity, increased age at first live birth, and limited breastfeeding are associated with an increased risk of breast cancer in the Chinese population, which is similar to Western women [8–10]. The one-child policy in China, it might have affected breast cancer risk by reducing the lifetime duration of breastfeeding [8]. Obesity and low levels of physical activity are both known as risk factors for breast cancer in Western countries. With the rapid growth of finance in China, these factors may also be risk factors for breast cancer in China [11]. Other factors, such as height, hormone replacement therapy, and family history, have also been regarded as risk factors for breast cancer in Chinese women [11, 12]. However, few studies have paid attention to psychological factors, such as the association between cancer diagnosis disclosure and the survival time of patients with breast cancer.

People display different attitudes towards life as their age grows. Some studies found that patients of different ages may display different emotional experiences. Although the incidence of breast cancer increases with age, younger patients may be more likely to suffer emotional distress than older patients. This relationship can be observed in patients ranging in age from 30 to over 80 years [13, 14]. Additionally, another study reported a nonsignificant correlation between age and affective distress by examining the above association 10 months after diagnosis [15]. Most studies had a limited sample size, which may affect the effectiveness of the conclusions.

Whether to fully inform patients of their cancer diagnosis has traditionally been a controversial topic. Advocates of concealing the condition to patients argue that patients who know their cancer diagnosis are liable to experience significant distress, which may lead to a worse prognosis [16]. Studies in Iran and Turkey showed that patients who knew their diagnosis were more likely to undergo depression, psychiatric morbidity and other negative emotions [17, 18]. On the other hand, patients who lack the awareness of their condition may have unrealistic optimism, which may lead to unfavourable behaviours and finally result in adverse health outcomes [19]. Previous studies showed that having an appropriate perspective of cancer status improved patients’ participation in the treatment and reduced their distress level, which helped them meet their psychological needs with self-esteem and respect [20]. In contrast, another study among elderly patients mentioned that not knowing their cancer condition results in an incomplete understanding of their diagnosis, which would hurt the trust relationship between these patients and physicians [21]. Based on a study of a total of 127 cancer patients and their caregivers, no significant difference was found before and after disclosing the cancer diagnosis to patients, while the quality of life could be improved with psychological care intervention [22]. We found that in patients with lung cancer, those who knew their cancer status had a better survival rate, while there are few studies about this issue in breast cancer patients [23].

The association between the diagnostic disclosure of breast cancer and the prognosis of patients was explored in this retrospective cohort study based on the baseline and long-term follow-up information of a large sample. The results may provide valuable evidence for clinical practice.

## Methods

The study was designed to determine the role that knowledge of the cancer diagnosis plays in the survival rate of patients with breast cancer by collecting information on patients with different backgrounds. Cancer disclosure was defined as informing patients of their cancer diagnosis. Patient knowledge of treatment, prognosis, and other relevant information was not considered.

### Participants

A total of 12,327 patients who were diagnosed with breast cancer between October 2002 and December 2016 were included in this study. All participants were registered at certified hospitals in Pudong New Area, Shanghai, China. This study followed the Helsinki Declaration of 1975. Written informed consent was obtained from the participants or their families.

### Data collection

The knowledge status of the cancer diagnosis of participants was collected at the time of study enrolment by the Shanghai Tumor Registry in accordance with Shanghai Tumor Report Card (Additional file 1). Information on demographics, breast cancer diagnosis, clinical stage and other relevant data was collected at the same time. The registration of cancer was conducted according to the criteria of the Chinese Guideline for Cancer Registration [24] and Cancer Incidence in Five Continents Volume IX [25]. The population data of cancer patients registered in the Shanghai Cancer Registry system were obtained from the Public Security Bureau of Pudong, Shanghai, China. The proportion of morphological verification (MV%), percentage of cancer cases identified with death certification only (DCO%), and mortality-to-incidence ratio (M/I) were 3 major measures to evaluate the primary exposures [26, 27]. We analysed the diagnosis based on the hospitalization medical record, which recorded whether the medical staff at that time had informed the patient of their cancer status. Centers for Disease Control staff regularly obtain information on cancer deaths from the Cause of Death Registration and Reporting System to determine the number of cancer deaths. Few patients were registered after death, and their knowledge status of cancer diagnosis was unclear. The survival data of patients with breast cancer were collected through the Centers for Disease Control and Prevention of the Pudong New Area, Shanghai. Community doctors followed up the patients by telephone calls or household surveys every 6 months, and regarded death as the primary outcome event. The follow-up ended in June 2017.

### Statistical analysis

The χ^2^ test was used to explore all categorical variables. One-way analysis of variance (ANOVA) was conducted to compare continuous variables. A life table was used to calculate the 3-year survival rate and 5-year survival rate and to compare the differences in the survival curves between subgroups. We included knowledge of cancer diagnosis status, sex, age, clinical stage, surgery history, diagnostic year and reporting hospital grade in the Cox proportional hazards regression model (forward stepwise, likelihood ratio test). The Cox proportional hazards regression model (forward stepwise, likelihood ratio test) with hazard ratios (HRs) and 95% confidence intervals (CIs) calculated was used to conduct multivariate analysis of factors influencing survival time. As shown in Additional file 2, the transformed Kaplan-Meier curves by the year of diagnosis displayed a graphical check for the proportionality of hazards. The assumptions of proportionality were met for the Cox models. Statistical Package for Social Sciences software (version 23.0, SPSS, Inc., Chicago, IL) was used to conduct all statistical analyses, and all tests were two-sided. *P* < 0.05 was defined as statistically significant.

### Propensity score matching (PSM)

We generated propensity scores (PSs) from a logistic regression model described by Rosenbaum and Rubin [28]. Sex, age, clinical stage, surgery history, the grade of reporting hospital and diagnostic year were included as covariates, which were selected into the model to optimize the matching procedure. In our retrospective study, each patient in the subgroup who knew of their diagnosis was 1:1 matched, with a calliper value of 0.02, to a corresponding patient in the subgroup who had no idea of their diagnosis by selecting the same PS for each pair. The 3-year survival rate and 5-year survival rate were calculated by a life table, and we compared the differences in the survival curves between the above subgroups.

## Results

### Baseline characteristics

A total of 12,327 patients with breast cancer were enrolled in this study. Table 1 shows the baseline characteristics of the participants. Among all 12,327 participants, 9466 (76.79%) patients were aware of their cancer diagnosis, and 2756 (22.36%) patients had no idea of their situation. There were 105 (0.85%) patients with unclear knowledge of their diagnosis. As shown in Table 1, there was no difference in sex composition between participants who knew their diagnosis and those who did not know (women: 99.26% vs. 99.24%, *P* = 0.904). Significant differences were found in age, clinical stage, surgery history and reporting hospital grade between patients who knew their diagnosis and those who did not (*P* < 0.05). A trend of informing the patients of their cancer diagnosis was also found by noticing the difference between different phases of the diagnostic year (when participants were diagnosed with breast cancer, 1: before 2006, 2: 2007–2011, 3: 2012–2016; linear-by-linear association: value = 4.232, *P* = 0.040). Patients who knew their diagnosis had a younger average age (55.97 ± 11.94 vs. 60.49 ± 14.20, *P* < 0.001), earlier clinical stage (stage 0–I: 31.94% vs. 22.28%, *P* < 0.001), higher surgery rate (56.20% vs. 49.24%, *P* < 0.001), and more recent diagnostic year (diagnosed from 2012 to 2017:40.78% vs. 40.50%, *P* = 0.001). In addition, patients registered and reported in higher grade hospitals were more likely to be informed of their diagnosis (high-grade hospital: 57.91% vs. 55.70%, *P* < 0.05).

**Table 1:** Demographic and clinical characteristics of participants, n(%)

Variable	Total sample (***N*** = 12,327)	Knowing status of cancer diagnosis			
		Did Know (***n*** = 9466)	Did not know (***n*** = 2756)	Unclear (***n*** = 105)	P^*^
**Sex**					
Male	93(0.74)	70(0.74)	21(0.76)	2 (1.90)	**0.904**
Female	12,234(99.26)	9396 (99.26)	2735(99.24)	103(98.10)	
**Average age**	57.10 ± 12.70	55.97 ± 11.94	60.49 ± 14.20	69.52 ± 15.10	**< 0.001**
< 35	380(3.08)	305(3.22)	74(2.68)	1(9.52)	**< 0.001**
35-	1564(12.69)	1258(13.29)	298(10.81)	8(6.60)	
45-	3821(31.00)	3137(33.14)	672(24.38)	12(7.62)	
55-	3488(28.30)	2762(29.18)	709(25.72)	17(16.19)	
65-	1789(14.51)	1289(13.62)	482(17.49)	18(17.14)	
≥ 75	1285(10.42)	715(7.55)	521(18.90)	49(46.67)	
**Clinical stage**					**< 0.001**
Stage 0- I	3641(29.54)	3024(31.94)	614(22.28)	3(2.86)	
Stage II	3700(30.02)	3037(32.08)	657(23.84)	6(5.71)	
Stage Ш	1135(9.21)	908(9.59)	220(7.98)	7(6.67)	
Stage IV	517(4.19)	358(3.78)	149(5.41)	10(9.52)	
Unclassified	3334(27.05)	2139(22.60)	1116(40.49)	79(75.24)	
**Surgery history**					**< 0.001**
Yes	6697(54.33)	5320(56.20)	1357(49.24)	20(19.05)	
No	5630(45.67)	4146(43.80)	1399(50.76)	85(80.95)	
**Diagnostic year** ^**a**^					
1: before 2006	3268(26.51)	2442(25.80)	802(29.10)	24(22.86)	**0.001**
2: 2007–2011	4074(32.83)	3163(33.41)	838(30.41)	73(69.52)	
3: 2012–2016	4985(40.44)	3861(40.78)	1116(40.50)	8(7.62)	
**Hospital grade**					**< 0.05**
Primary grade hospital	166(1.35)	103(1.09)	45(1.63)	18(17.14)	
Middle grade hospital	5088(41.28)	3881(41.00)	1176(42.67)	31(29.52)	
High grade hospital	7073(57.38)	5482(57.91)	1535(55.70)	56(53.33)	

^*^ patients who knew diagnosis vs. patients who did not know diagnosis

^a^ Diagnostic year 1 means those being diagnosed before 2006, 2 means those being diagnosed from 2007 to 2011, 3 mean those being diagnosed from 2012 to 2016

### Univariate analysis of factors influencing the survival time of patients with breast cancer

**Fig. 1:**
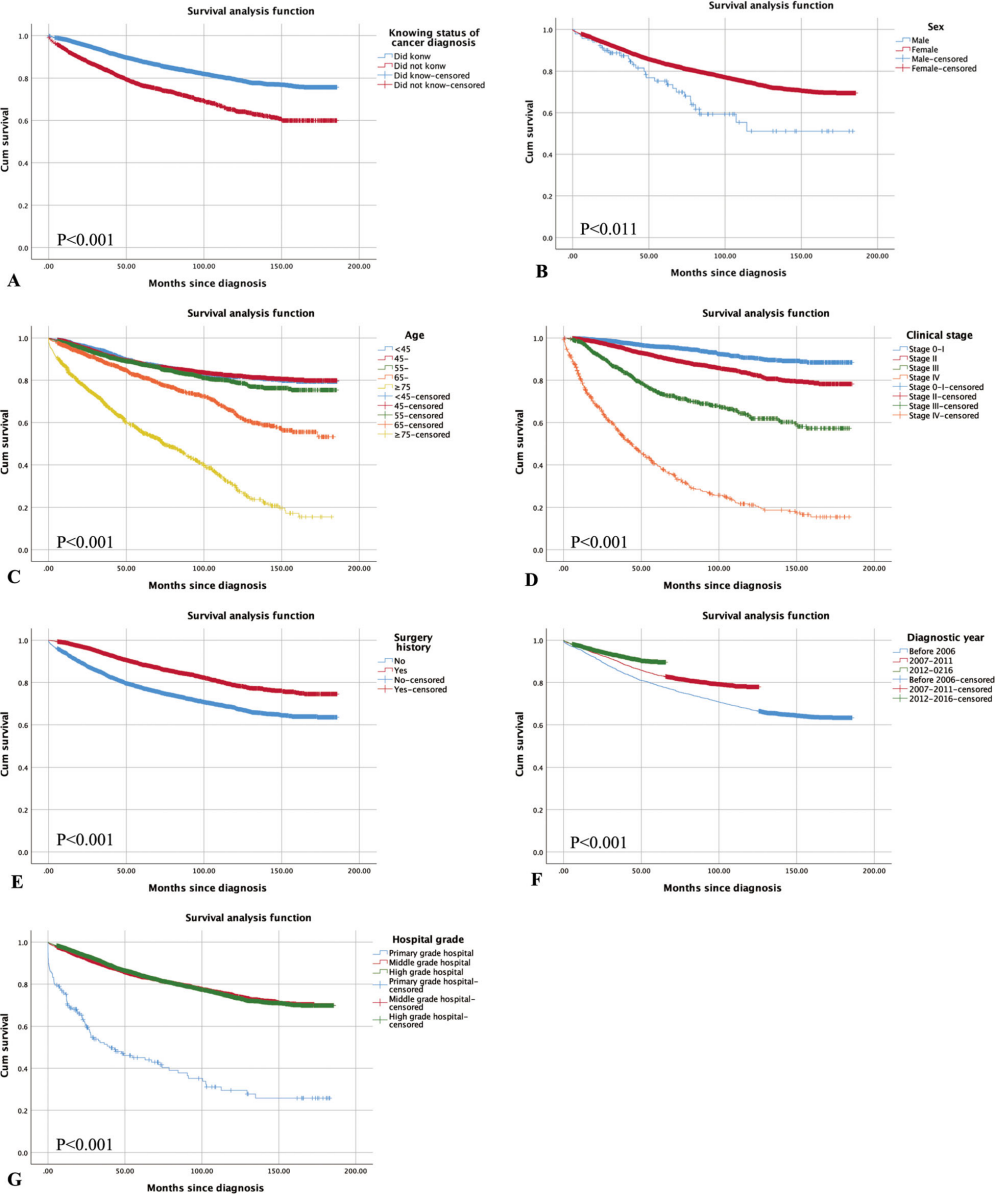
Survival of breast cancer patients with different characteristics. **A**, the patients knew their cancer diagnosis vs. those did not know; **B**, the male patients vs. the female patients; **C**, the patients younger than 45 vs. those aging from 45-55 vs. those aging from 55-65 vs. those aging from 65-75 vs. those older than 75; **D**, the patients at stage 0-I vs. those at stage II vs. those at stage III vs. those at stage IV; **E**, the patients without surgery history vs. those with surgery history; **F**, the patients diagnosed before 2006 vs. those diagnosed between 2007-2011 vs. those diagnosed between 2012-2016; **G**, the patients reported from primary grade hospital vs. those reported from middle grade hospital vs. those reported from high grade hospital

Altogether, 2152 (17.24%) deaths occurred among the 12,327 registered patients during the 14-year median follow-up. The 3-year survival rate and 5-year survival rate of our participants were 0.86 and 0.81, respectively. As shown in Table 2 and Fig. 1, participants with different characteristics had significant differences in prognosis. The 3-year survival rate and 5-year survival rate of patients who knew their diagnosis were both longer than those who did not know (0.89 vs. 0.79, 0.85 vs. 0.73, *P* < 0.001). In addition, it was found that female sex (*P* = 0.011), younger age (< 35 years: *P* < 0.001), earlier clinical stage (*P* < 0.001), higher rate of surgery (*P* < 0.001), being diagnosed more recently (*P* < 0.001) and being reported from higher grade hospitals (*P* < 0.001) contributed to a better survival rate.

**Table 2:** Survival of breast cancer patients with different characteristics

Variable	Death number/Total number	3-year survival rate	5-year survival rate	P^*^
**Knowing status of cancer diagnosis** ^**a**^				**< 0.001**
Did know	1446/9466	0.89	0.85	
Did not know	778/2756	0.79	0.73	
**Sex**				**0.011**
Male	29/93	0.78	0.70	
Female	2300/12234	0.86	0.81	
**Age (years)**				**< 0.001**
< 45	259/1944	0.90	0.87	
45-	543/3821	0.90	0.86	
55-	473/3488	0.89	0.86	
65-	408/1789	0.85	0.78	
≥ 75	646/1285	0.62	0.51	
**Clinical stage** ^**b**^				**< 0.001**
Stage 0- I	197/3640	0.97	0.95	
Stage II	480/3700	0.92	0.88	
Stage Ш	316/1136	0.78	0.70	
Stage IV	358/517	0.44	0.33	
**Surgery history**				**< 0.001**
Yes	890/6697	0.91	0.87	
No	1439/5630	0.80	0.75	
**Diagnostic year** ^**c**^				
1: before 2006	1164/3268	0.82	0.76	**< 0.001**
2: 2007–2011	815/4074	0.86	0.82	
3: 2012–2016	350/4985	0.91	0.89	
**Hospital grade**				**< 0.001**
Primary grade hospital	95/166	0.47	0.41	
Middle grade hospital	972/5088	0.86	0.82	
High grade hospital	1262/7073	0.87	0.82	

^*^overall comparison of survival curves in subgroups

^a^ patients with unclear knowing status of cancer diagnosis were not included

^b^ patients with unclassified clinical stage were not included

^c^ Diagnostic year 1 means those being diagnosed before 2006, 2 means those being diagnosed from 2007 to 2011, 3 mean those being diagnosed from 2012 to 2016

### Stratified analysis of the impact of cancer diagnosis knowledge on survival time in patients with breast cancer

As shown in Table 3 and Additional file 3, participants were stratified by sex, age, clinical stage, surgery history, diagnostic year and grade of reporting hospital to explore the relationship between awareness of cancer diagnosis and prognosis. Except for the subgroups of male patients (*P* = 0.103) and stage Ш patients (*P* = 0.265), patients who knew their cancer diagnosis displayed a better survival rate than those who did not (*P* < 0.05).

**Table 3:** Relationship between cancer awareness and survival time of breast cancer patients by stratified analysis

Stratified factors	3-year survival rate		5-year survival rate		P^*^
	Did know cancer diagnosis	Did not know cancer diagnosis	Did know cancer diagnosis	Did not know cancer diagnosis	
**Sex**					
Male	0.85	0.66	0.76	0.58	**0.103**
Female	0.89	0.79	0.85	0.73	**< 0.001**
**Age (years)**					
< 45	0.92	0.87	0.88	0.82	**< 0.001**
45-	0.91	0.86	0.87	0.82	**< 0.001**
55-	0.91	0.85	0.87	0.81	**< 0.001**
65-	0.89	0.79	0.82	0.73	**< 0.001**
≥ 75	0.70	0.56	0.60	0.45	**< 0.001**
**Clinical stage** ^**a**^					
Stage 0- I	0.97	0.95	0.96	0.91	**< 0.001**
Stage II	0.93	0.89	0.89	0.85	**0.001**
Stage Ш	0.79	0.76	0.71	0.69	**0.265**
Stage IV	0.47	0.40	0.34	0.31	**0.005**
Unclassified	0.84	0.70	0.79	0.63	**< 0.001**
**Surgery history**					
Yes	0.92	0.87	0.88	0.82	**< 0.001**
No	0.85	0.70	0.81	0.63	**< 0.001**
**Diagnostic year** ^**b**^					
1: before 2006	0.85	0.73	0.80	0.67	**< 0.001**
2: 2007–2011	0.90	0.80	0.86	0.75	**< 0.001**
3: 2012–2016	0.93	0.84	0.92	0.81	**< 0.001**
**Hospital grade**					
Primary grade hospital	0.60	0.36	0.52	0.36	**0.034**
Middle grade hospital	0.89	0.78	0.86	0.71	**< 0.001**
High grade hospital	0.89	0.81	0.85	0.75	**< 0.001**

^*^ overall comparison of survival curves in subgroups

^a^ patients with unclear knowing status of cancer diagnosis were not included

^b^ Diagnostic year 1 means those being diagnosed before 2006, 2 means those being diagnosed from 2007 to 2011, 3 mean those being diagnosed from 2012 to 2016

### Multivariate analysis of factors influencing the survival time of patients with breast cancer

Knowledge of cancer diagnosis status, age, surgery history, reporting hospital grade, diagnostic year and clinical stage were independent influencing factors of the survival time of patients with breast cancer (*P* < 0.001, Table 4). The results showed that not knowing of the cancer diagnosis was significantly associated with a poor prognosis compared to knowing (HR, 1.405, 95% CI, 1.285–1.537, *P* < 0.001). Having surgery contributed to a better survival (HR, 0.647,95% CI, 0.594–0.706; *P* < 0.001), while age was a risk factor for survival time (HR, 1.434,95% CI, 1.285–1.537; *P* < 0.001). Compared to patients from primary grade hospitals, patients from higher grade hospitals had a better survival rate (HR, 0.457, and 0.478, respectively; *P* < 0.001). The more recently the patients were diagnosed, the longer they were likely to survive (HR, 0.802, and 0.649, respectively; *P* < 0.001, Table 4). In addition, compared to stage 0- I patients, more advanced stage patients had a poorer prognosis (HR, 2.085, 4.988, and 13.953, respectively; *P* < 0.001, Table 4).

**Table 4:** Factors influencing survival time of breast cancer patients by Cox proportional hazard regression model (*n* = 12,327)

Variable	B	SE	HR (95% CI)	***P***
**Knowing status of cancer diagnosis**				
Did know			1.00	**< 0.001**
Didn’t know	0.34	0.05	1.41(1.29–1.54)	**< 0.001**
Unclear	1.61	0.11	5.02(4.05–6.22)	**< 0.001**
**Age**	0.36	0.02	1.43(1.39–1.48)	**< 0.001**
**Surgery history**	−0.44	0.04	0.65(0.59–0.71)	**< 0.001**
**Hospital grade**				
Primary grade hospital			1.00	**< 0.001**
Middle grade hospital	−0.78	0.11	0.46(0.37–0.57)	**< 0.001**
High grade hospital	−0.74	0.11	0.48(0.39–0.60)	**< 0.001**
**Diagnostic year** ^**a**^				
1: before 2006			1.00	**< 0.001**
2: 2007–2011	−0.22	0.05	0.80(0.73–0.88)	**< 0.001**
3: 2012–2016	− 0.43	0.07	0.65(0.59–0.71)	**< 0.001**
**Clinical Stage**				
Stage 0- I			1.00	**< 0.001**
Stage II	0.74	0.09	2.09(1.77–2.46)	**< 0.001**
Stage Ш	1.61	0.09	4.99(4.17–5.96)	**< 0.001**
Stage IV	2.64	0.09	13.95(11.70–16.64)	**< 0.001**
Unclassified	1.37	0.08	3.94(3.37–4.60)	**< 0.001**

*HR* hazard ratio, *CI* confidence interval

^a^ Diagnostic year 1 means those being diagnosed before 2006, 2 means those being diagnosed from 2007 to 2011, 3 mean those being diagnosed from 2012 to 2016

### The impact of cancer diagnosis knowledge on survival time in patients with breast cancer after PSM

**Fig. 2:**
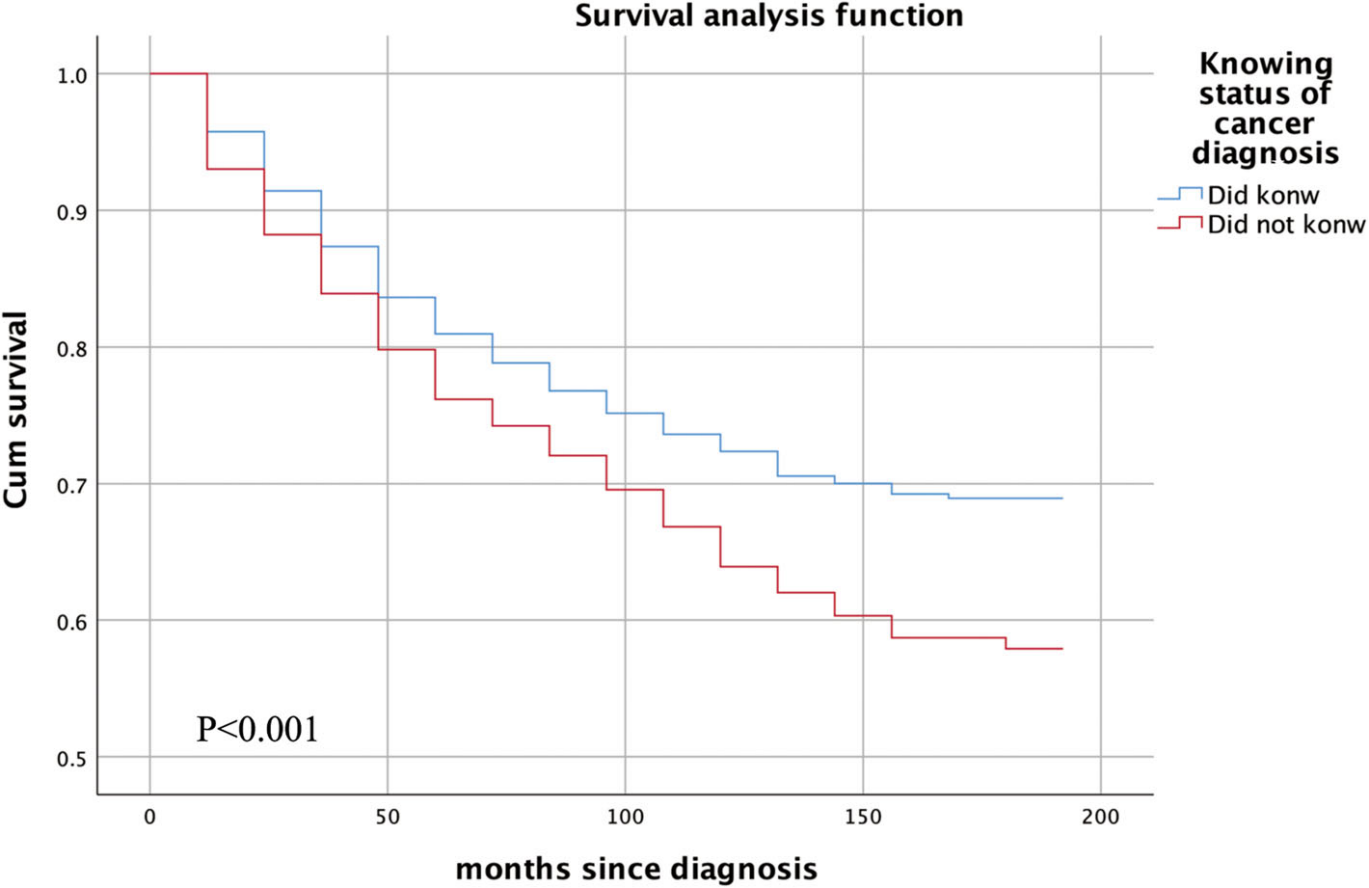
Difference of survival time between patients knowing their diagnosis and patients not of breast cancer patients after PSM. A, the patients knew their cancer diagnosis vs. those did not know

After PSM, 2694 pairs (2694 patients who knew their diagnosis and 2694 patients who did not) were selected from 9466 patients who knew their diagnosis and 2756 patients who did not. Both the 3-year survival rate and 5-year survival rate of patients who knew their diagnosis were longer than those who did not know (0.84 vs. 0.80, 0.79 vs. 0.74; *P < 0.001,* Table 5, Fig. 2).

**Table 5:** Survival of breast cancer patients knowing or not knowing diagnosis, before and after propensity score matching

Variable	Before PSM				After PSM			
	Death number/Total number	3-year survival rate	5-year survival rate	P^*^	Death number/ Total number	3-year survival rate	5-year survival rate	P^*^
**Knowing status of cancer diagnosis** ^**a**^				**< 0.001**				**< 0.001**
Did know	1446/9466	0.89	0.85		700/2694	0.84	0.79	
Did not know	778/2756	0.79	0.73		597/2694	0.80	0.74	

^*^overall comparison of survival curves in subgroups

^a^ patients with unclear knowing status of cancer diagnosis were not included

## Discussion

According to our long-term follow-up research of a large sample, disclosure of patients’ diagnosis was found to be a protective factor for the longer survival time of patients with breast cancer through univariate and multivariate analyses. Age, clinical stage, surgery history and diagnosis year were also associated with patient survival time.

The disclosure of cancer diagnosis to patients with breast cancer has always been a contentious topic worldwide. Owing to the different cultural backgrounds of different countries, the opinions on this topic are varied. A survey indicated that in the United Kingdom, almost all patients wanted to be aware of their diagnosis, while in Asian culture, physicians and family members may worry more about whether to inform patients [29]. Moreover, there was a change in attitude when the hypothetical diagnosis changed from the initial stage to the terminal stage. The percentage of those who wanted to reveal the diagnosis to patients decreased significantly (from 87.5 to 40.5%) [30]. The reasons why physicians and family members hesitate to disclose this information to patients may include the psychological impact and pain from treatment patients undergo, especially the loss of physical integrity. It has been suggested that losing a breast by mastectomy could bring about severe mental impairments resulting from body image, female identity, self-worth, social interactions and so on [31].

In this study, we found that the popularization of informing patients of their diagnosis is increasing year by year, and the disclosure of patients’ diagnosis is an independent protective factor for patients with breast cancer to prolong their survival time. Patients not knowing of their real condition may have unrealistic optimism, which may lead to an unhealthy lifestyle, making their condition worse. In contrast, having a clear perspective of their cancer status may lead to a healthier way of life. Informing patients with breast cancer as early as possible assists them in obtaining precise knowledge of themselves. Moreover, there are many strategies to help patients cope with their emotional distress such as psychological care and breast reconstruction, which have already had some effectiveness [32, 33].

In the male patient subgroup, diagnostic disclosure was not linked with survival time. Male breast cancer is uncommon, and only 93 (0.75%) male patients were involved in our study. Similarly, there were 2470 (0.98%) men with breast cancer and 252,710 women with breast cancer in 2017 in the USA [34]. Since most data for breast cancer research are from female patients, men tend to be diagnosed with breast cancer at a later age than women since most treatment and diagnostic decisions are made based on female patients’ data [35]. In addition, breast cancer appeared in female patients more frequently, therefore, male patients may undergo a special perceptual experience. Because of the sex stereotype, male patients knowledgeable of their diagnosis were more likely to have a high level of cancer-specific distress and depressive symptoms [36]. More research may be needed on the psychological state of this particular and rare group.

Our study found that disclosure of cancer status and other factors, such as female sex, younger age, earlier clinical stage, surgery history, and more recent diagnostic year, were significantly related to better survival. As shown in Additional file 4, patients without surgery history were less likely to be aware of their cancer condition (did not know: 25.23% vs. 20.32%), as were patients with a later clinical stage (stage IV: 5.75% vs. 2.88%), those with an earlier diagnostic year (diagnosis before 2006: 35.68% vs. 18.80%) and those with a lower hospital grade (primary and middle grade hospitals: 1.85% vs. 0.92 and 45.93% vs. 37.36%, *P* < 0.05). There are several possible reasons for this result. Patients who had no idea of their diagnosis could hardly have a clear understanding of their treatment, which decreased their surgery rate. Patients at later clinical stages such as stage IV, were not advocated to undergo a surgery because of their conditions. In addition, the conditions for performing surgery in primary hospitals may not have been complete in earlier times, which may also limit the chances of surgery. A more recent diagnostic year predicts a shorter time of cancer to development and more advanced treatment. Meanwhile, more patients were told of their cancer status in the more recent diagnostic years. Therefore, patients with these conditions may have a better prognosis.

In our study, we used a narrow definition of cancer disclosure, focusing only on whether the patient was informed of their cancer diagnosis and not considering the patient’s knowledge of treatment, prognosis, and other relevant information. We did not obtain detailed information about the treatment that participants received, which may have influenced the final results of the participants. Because of challenges in data collection and uncertainty, some potential factors, such as the psychological condition of patients, education level and income, were not included. In future research, patients might be concerned about their own conditions; however, the decision the patients’ families made at the first diagnosis may reflect the patients’ characteristics to some extent. Therefore, we regarded their diagnosis as concealed if they did not know their own condition when they were enrolled in our study. Since our follow-up lasted for a long time, some participants might have known about their condition after discharge from the hospital because of personal perception or other reasons. However, this phenomenon did not mean that our classification of the status of whether the diagnosis was known was inappropriate. According to the Shanghai Cancer Patient Follow-up Manual, the information of patients who were unaware of their cancer status would be obtained from follow up by their families, and patients who were aware of their diagnosis would provide the information by themselves. Since we made the first classification according to the hospitalization medical record, we recorded whether the medical staff at that time had informed the patient of their cancer status. The hospitalization medical record was given certain legal validity, which means that we could not change the classification due to this unsteady state. The establishment of a prospective cohort and further research on the basis of the current study will help solve this problem.

The Cox regression analysis showed that 105 patients with uncertain knowledge status of cancer diagnosis had a 5 times higher risk of death than those who knew about their cancer diagnosis. These 105 patients were older (average age: 69.52 ± 15.10 years, ≥75 years: 46.67%), and mostly had an unclassified clinical stage (75.24%). Most of them did not undergo surgery (80.95%) and were diagnosed before 2011 (92.38%) (Table 1). Older patients have a greater dependency on their guardians, which made their information less clear to medical staff. Patients with an unclassified stage hardly underwent surgeries, and some of them had only been hospitalized for a short time at a late clinical stage (Additional file 5). In addition, being diagnosed earlier and attending a hospital with a lower grade may mean an incomplete registration system for personal information and outdated treatment. These factors may contribute to a higher risk of death.

This retrospective cohort study provides clear evidence to promote diagnostic disclosure to breast cancer patients through the long-term follow-up of a large sample size, which hopefully offers a new direction in clinical practice.

Our study suggested that an increasing number of medical staff and patients’ families are more willing to disclose the truth to patients according to our analysis on diagnostic year, which implies that knowledge of the cancer status predicts a better prognosis and a longer survival time than not knowing. Positive evidence suggests that disclosing the diagnosis to patients is better for the survival of patients with breast cancer. Previous studies showed that most patients were ready to have sufficient knowledge of their diagnosis, while the majority of medical staff and families were not [37] The reason for this phenomenon may be that it is a difficult task to reveal the cancer diagnosis to patients, which would make physicians feel uncomfortable and unprepared [38, 39]. With the consent right becoming ever more common, the debate about whether to tell patients about their diagnosis would be replaced by how and when to tell them about their diagnosis. Existing guidelines for breaking bad news based on expert opinion are available [40]. However, some guidelines were not completely derived from empirical data [41]. Therefore, formal guidelines for breaking bad news must be made. Furthermore, oncological care for patients is also needed to help them maintain a positive attitude towards their cancer status and overcome their emotional distress [42].

In this study, we did not obtain the detailed treatments received by the participants, which is a major restrictive factor. Different treatments might result in different prognosis and make variety impacts on patients. Because of challenges in data collection and uncertainty, some potential factors were not included, such as household income, education level and psychological condition of patients, which are also study limitations.

## Conclusions

Based on the long-term follow-up of a large sample, the popularization of the disclosure of the cancer diagnosis to patients has increased over time, which may contribute to a longer survival time in patients with breast cancer. Even though being told of their cancer status may arouse emotional distress in patients, with more adequate information on their condition and better skills of medical staff to disclose this information, patients would benefit from knowing this information in the long term. Formal guidelines of medical staff to reveal cancer diagnosis and education for patients’ families of psychological care need further complement in clinical practice. Our findings provide further evidence supporting the disclosure of the cancer diagnosis to patients. Future prospective studies are needed to validate these findings.

## Supplementary Information

**Additional file 1.** Shanghai Cancer Report Card.

**Additional file 2.** Plot to check proportional hazards assumption of the Cox model.

**Additional file 3.** Survival time of breast cancer patients knowing and not knowing diagnosis by stratified analysis.

**Additional file 4.** Demographic and clinical characteristics of patients with or without surgery history.

**Additional file 5.** Demographic and clinical characteristics of participants with unclassified clinical stage.

## Data Availability

The datasets used and analysed during the current study are available from the corresponding author on reasonable request.

## Abbreviations

PSM: Propensity score matching
NCI: National Cancer Institute
SEER: Surveillance, Epidemiology, and End Results
MV%: Morphological verification
DCO%: Death certification only
M/I: Mortality-to-incidence ratio
ANOVA: One-way analysis of variance
HRs: Hazard ratios
CIs: Confidence intervals
PSs: Propensity scores
