# Supplementary material for: Knowledge of a cancer diagnosis is a protective factor for the survival of patients with breast cancer: a retrospective cohort study
Source: BMC Cancer. 2021 Jun 27;21:739. doi: 10.1186/s12885-021-08512-1 (PMC8237449; doi:10.1186/s12885-021-08512-1)
Supplement: Supplementary file 1 — Additional file 1. Shanghai Cancer Report Card. [file 12885_2021_8512_MOESM1_ESM.pdf]

Report area: Pudong New Area   Number: ___________   ICD code: ___________   ICD-O code:______________

(The followings are filled by the hospitals)

Shanghai Tumor Report Card

The diagnosis has been informed to the patient ☐ 1) Yes ☐ 2) No

Name: ___________ Sex: ___________ Nationality: ___________ Identity Card Number:___________

Telephone: ___________ Date of birth:______________________

Employment status: ___________ Occupation: ___________ Employer organization:

Domicile address:____

Residential Address:____

Column of corrected diagnosis

(Fill in when the original report was diagnosed incorrectly)

Original report:___________

Date of original report: ___________

(Please filled in if the domicile address and residential address is different.)

Outpatient ID: ___________ Hospitalized ID:______________________

(If it is a secondary tumor, please indicate the primary site as detailed as possible)

Diagnosis (Location): ___________ The location of tumor ☐ Left ☐ Right ☐ Both ☐ Unknown

Pathological diagnosis: ____________________

Pathology Number: ______________________

Clinical Stage: T_____N_____M_____ Stage 0 Stage I Stage II Stage III Stage IV Unclassified

First diagnostic time:____________ Report hospital:

Report doctor:_________________ Date of report:

Date of death:_________________ Reason of death:

Basis for Diagnosis: (click √ in ☐)

Clinical	1	Pathology (Secondary)	6
X-ray ☐ US ☐ CT ☐ Endoscopy ☐ MRI ☐	2	Pathology (Original)	7
Surgery ☐ Autopsy ☐ (Without pathology)	3	Autopsy ☐ (With pathology)	8
Biochemical ☐ Immunity ☐	4	Unknown	9
Blood tablets ☐ Cytology ☐	5	Illness diagnosed after death	10

First visit content: Name of person accepted the first visit: ☐ 1. Self ☐ __________________ Date of the first visit: ______________________

Height: __________________ Weight: __________________ Signature of First Visiting Doctor: __________________

Smoking status: ☐ 1. Smoking every day ☐ 2. Smoking but not every day ☐ 3. Used to smoke but quit now ☐ 4. Never smoke

Passive smoking place:

☐ 1. At home ☐ 2. Working places ☐ 3. Restaurants ☐ 4. Entertainment places ☐ 5. Public transportation and its waiting room ☐ 6. Other places

The age when started smoking every day: ____________ Average number of cigarettes smoked per day: _________________ Age to quit smoking: ____________

Date of first symptoms:__________________ Date of first visit to hospital: __________________ Current Condition: 1) Stable 2) Better 3) Worse

Previous Treatment: ☐ 1) Surgery ☐ 2) Chemotherapy ☐ 3) Radiotherapy ☐ 4) Traditional Chinese Medicine ☐ 5) Immunity ☐ 6) Intervention ☐ 7) Analgesic ☐ 8) Untreated ☐ 9) Other treatments

Name of hospital accepting treatments from:

Hospital giving surgery: __________________;__________________;__________________

Hospital giving chemotherapy: __________________;__________________;__________________

Hospital giving radiotherapy: __________________;__________________;__________________

Name of hospital giving the first surgery: __________________ Date of the first surgery: __________________

Character of the first surgery: ☐ 1) Cure ☐ 2) Palliative ☐ 3) Exploration Hospitalized ID:_______________________ Pathology Number: _________

Metastasis or not: ☐ 1) Yes ☐ 2) No Location of metastasis: _____________________

Relapse or not: ☐ 1) Yes ☐ 2) No Date of relapse: _____________________

Family history of tumor: ☐ 1) Yes ☐ 2) No If Yes, the relationship: __________________ the kind of tumor: __________________

Guidance: ☐ 1) Supervised follow-up ☐ 2) Medicine ☐ 3) Diet ☐ 4) Exercise ☐ 5) Treated at home ☐ 6) Treated in hospital ☐ 7) Visit patients at home

Kanorfsky performance score, KPS: __________________ Remarks:

Date of revocation of management: __________________ Reason of revocation of management: ☐ 1) Misdiagnosis ☐ 2) Refusal to visit ☐ 3) Living in other places ☐ 4) Domicile being changed to other places ☐ 5) Other reasons

Date of death: __________________ Reason of death: ☐ 1) Tumor ☐ 2) Non-tumor __________________

Place of death: ☐ 1) Hospital ward ☐ 2) Emergency room ☐ 3) At home ☐ 4) Out of town ☐ 5) Family ward ☐ 6) Nursing home ☐ 7) Died before arriving hospital ☐ 8) Other places

Follow-up record

Date	Name of visit doctor	Name of patient to visit	Items										KPS	Remarks
			Treatment situation	Relapse or not	Number of relapses	Date of relapse	Metastasis or not	Location of metastasis	Current condition	Weight(kg)	Smoke or not	Guidance		

Karnofisky performance score, KPS

100	Normal	40	Loses the ability to take care of himself or herself, and needs special care and help
90	Almost normal but have mild symptoms and signs	30	Loses the ability to take care of himself or herself completely
80	Barely normal and have some symptoms and signs	20	Seriously ill, and requires hospitalization and positive treatment
70	Able to take care of himself or herself, but unable to maintain normal work and life	10	Critically ill, near death
60	Barely able to take care of himself or herself but sometimes needs care and help	0	Death
50	Needs care and help		
