# Supplementary material for: Knowledge of a cancer diagnosis is a protective factor for the survival of patients with breast cancer: a retrospective cohort study
Source: BMC Cancer. 2021 Jun 27;21:739. doi: 10.1186/s12885-021-08512-1 (PMC8237449; doi:10.1186/s12885-021-08512-1)
Supplement: Supplementary file 2 — Additional file 2. Plot to check proportional hazards assumption of the Cox model. [file 12885_2021_8512_MOESM2_ESM.pdf]

Additional file 2

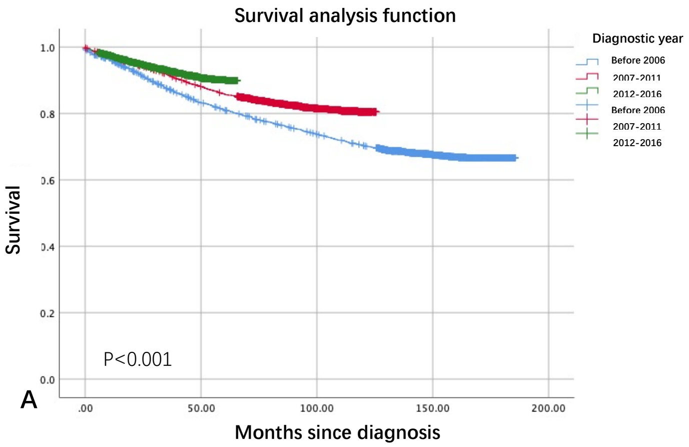

Additional file 2. Plot to check proportional hazards assumption of the Cox model.
