# Supplementary material for: Knowledge of a cancer diagnosis is a protective factor for the survival of patients with breast cancer: a retrospective cohort study
Source: BMC Cancer. 2021 Jun 27;21:739. doi: 10.1186/s12885-021-08512-1 (PMC8237449; doi:10.1186/s12885-021-08512-1)
Supplement: Supplementary file 3 — Additional file 3. Survival time of breast cancer patients knowing and not knowing diagnosis by stratified analysis. [file 12885_2021_8512_MOESM3_ESM.pdf]

Additional file 3

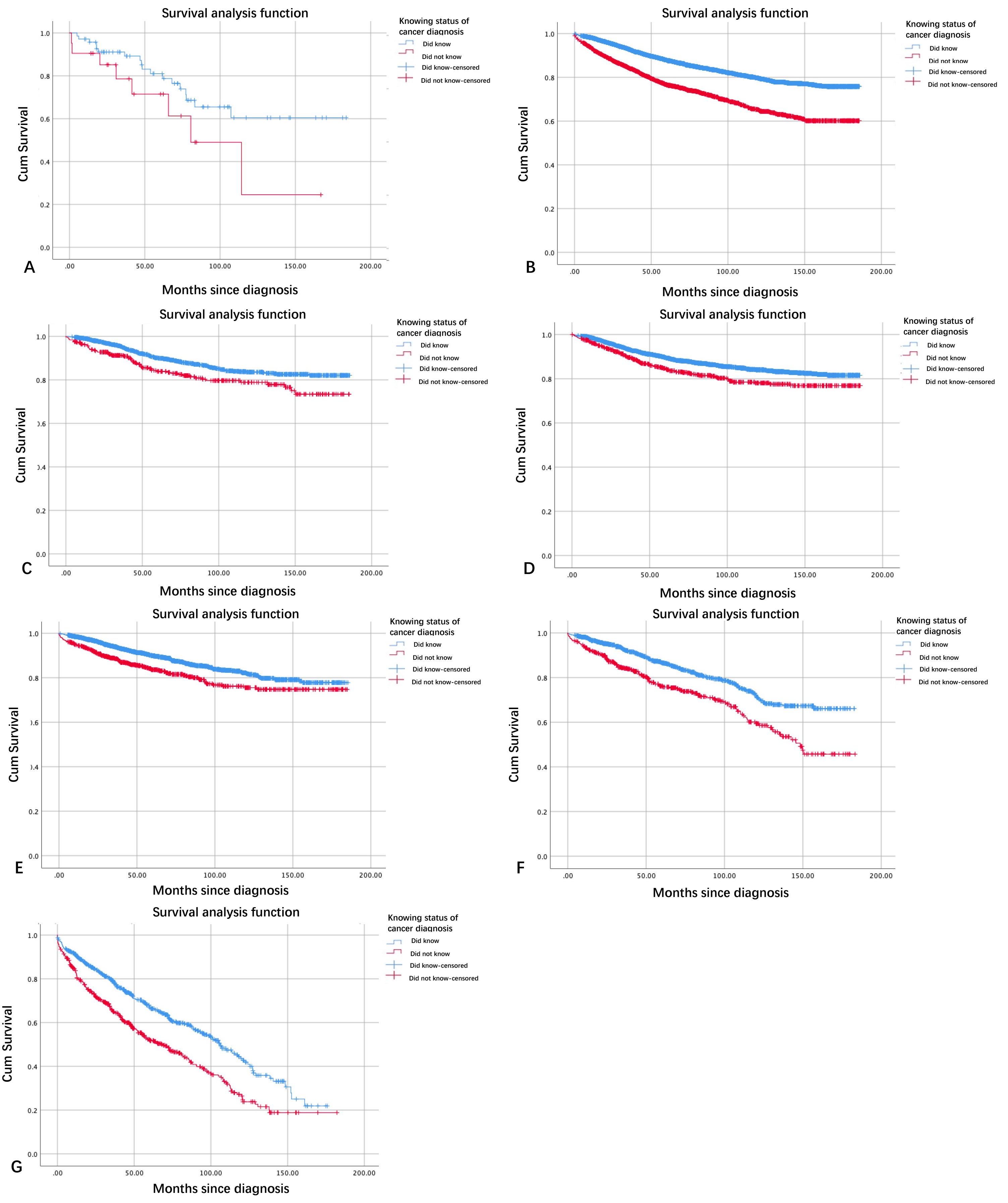

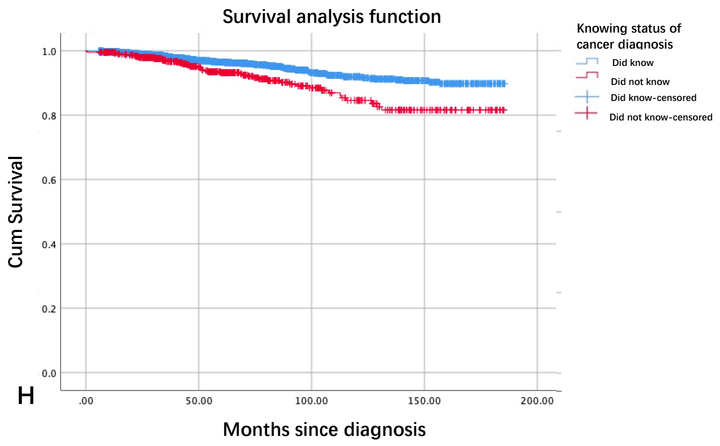
Survival analysis function
Knowing status of cancer diagnosis
Did know
Did not know
Did know-censored
Did not know-censored
Cum Survival
1.0
0.8
0.6
0.4
0.2
0.0
.00
50.00
100.00
150.00
200.00
Months since diagnosis
H

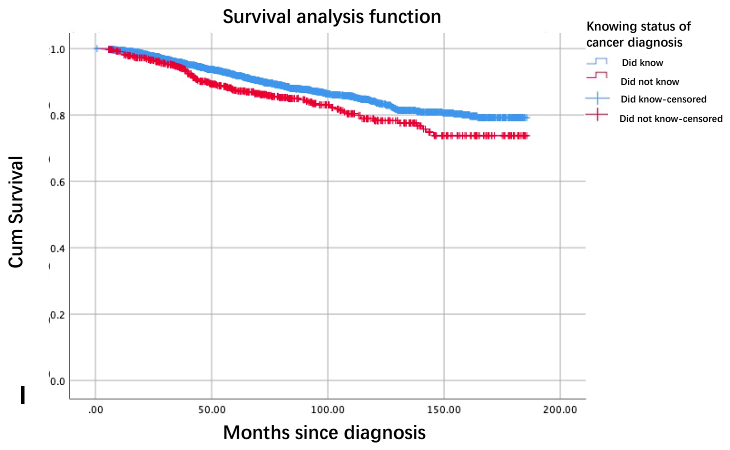
Survival analysis function
Knowing status of cancer diagnosis
Did know
Did not know
Did know-censored
Did not know-censored
Cum Survival
1.0
0.8
0.6
0.4
0.2
0.0
.00
50.00
100.00
150.00
200.00
Months since diagnosis
I

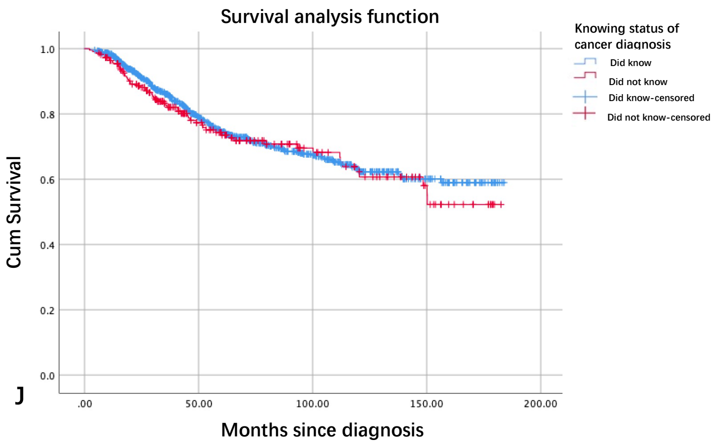
Survival analysis function
Knowing status of cancer diagnosis
Did know
Did not know
Did know-censored
Did not know-censored
Cum Survival
1.0
0.8
0.6
0.4
0.2
0.0
.00
50.00
100.00
150.00
200.00
Months since diagnosis
J

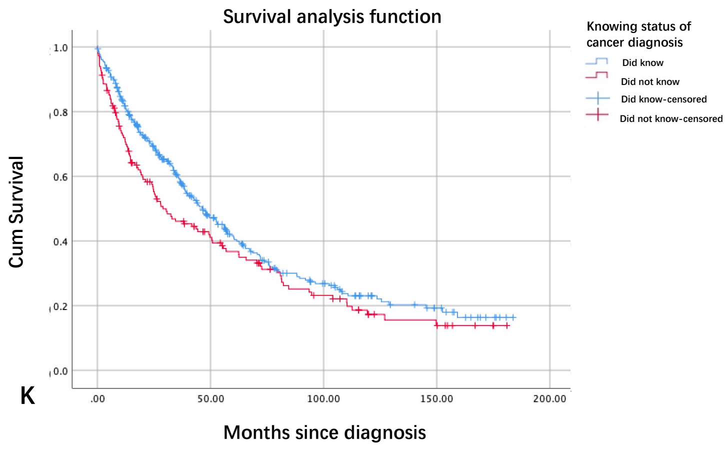
Survival analysis function
Knowing status of cancer diagnosis
Did know
Did not know
Did know-censored
Did not know-censored
Cum Survival
1.0
0.8
0.6
0.4
0.2
0.0
.00
50.00
100.00
150.00
200.00
Months since diagnosis
K

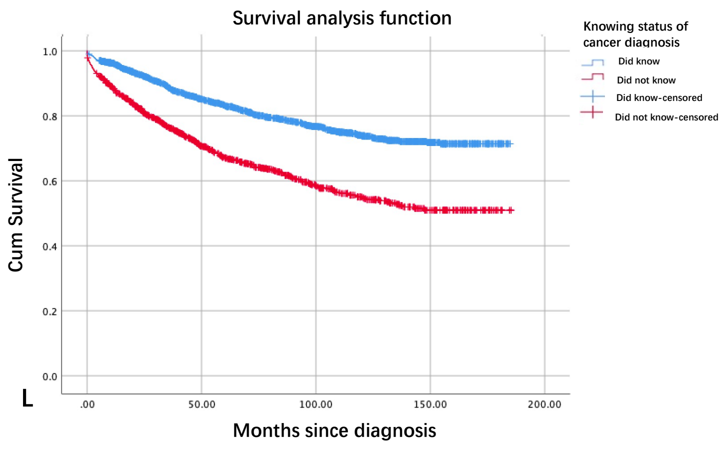
Survival analysis function
Knowing status of cancer diagnosis
Did know
Did not know
Did know-censored
Did not know-censored
Cum Survival
1.0
0.8
0.6
0.4
0.2
0.0
.00
50.00
100.00
150.00
200.00
Months since diagnosis
L

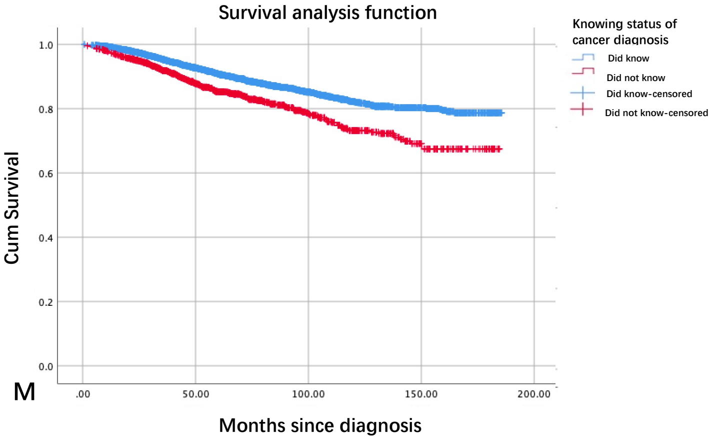
Survival analysis function
Knowing status of cancer diagnosis
Did know
Did not know
Did know-censored
Did not know-censored
Cum Survival
1.0
0.8
0.6
0.4
0.2
0.0
.00
50.00
100.00
150.00
200.00
Months since diagnosis
M

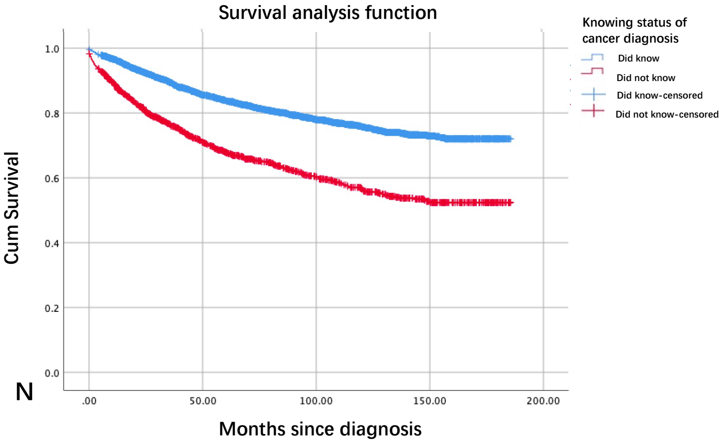
Survival analysis function
Knowing status of cancer diagnosis
Did know
Did not know
Did know-censored
Did not know-censored
Cum Survival
1.0
0.8
0.6
0.4
0.2
0.0
.00
50.00
100.00
150.00
200.00
Months since diagnosis
N

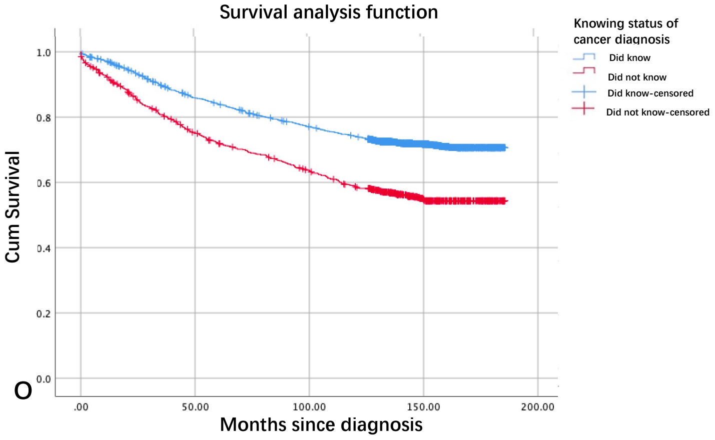
Survival analysis function
Knowing status of cancer diagnosis
Did know
Did not know
Did know-censored
Did not know-censored
Cum Survival
1.0
0.8
0.6
0.4
0.2
0.0
.00
50.00
100.00
150.00
200.00
Months since diagnosis
O

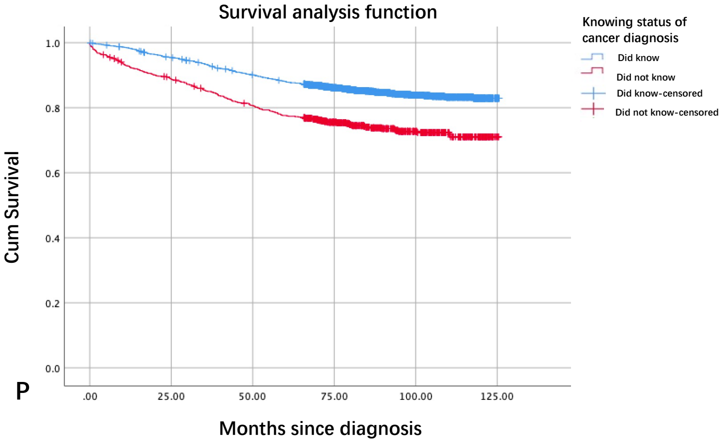
Survival analysis function
Knowing status of cancer diagnosis
Did know
Did not know
Did know-censored
Did not know-censored
Cum Survival
1.0
0.8
0.6
0.4
0.2
0.0
.00
25.00
50.00
75.00
100.00
125.00
Months since diagnosis
P

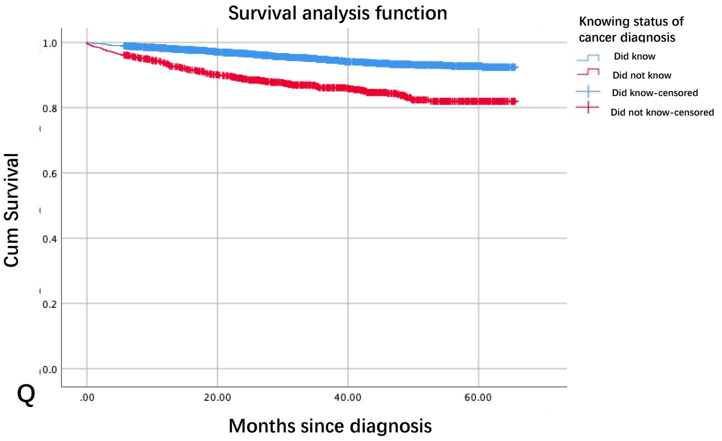
Survival analysis function
Knowing status of cancer diagnosis
Did know
Did not know
Did know-censored
Did not know-censored
Cum Survival
1.0
0.8
0.6
0.4
0.2
0.0
.00
20.00
40.00
60.00
Months since diagnosis
Q

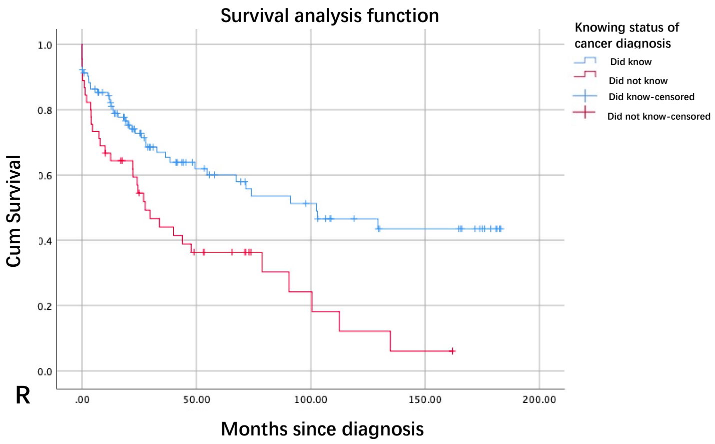

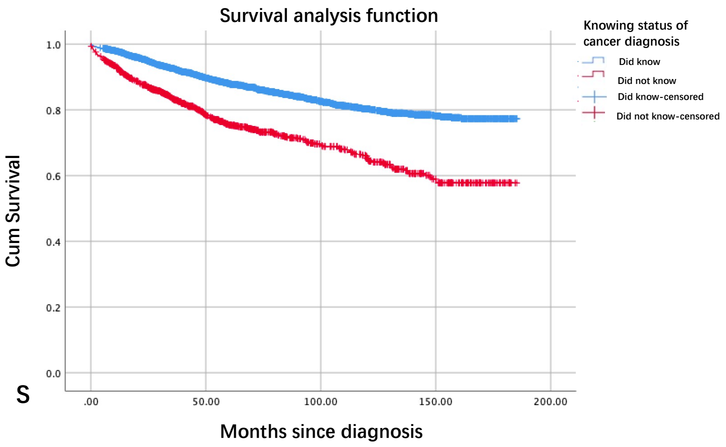

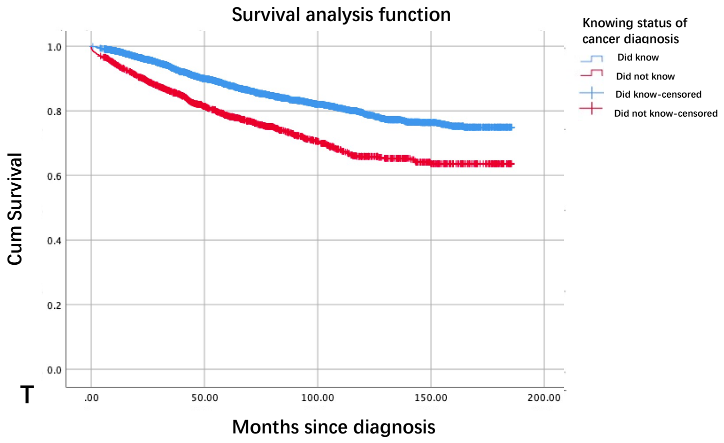

Additional file 3. Survival time of breast cancer patients knowing and not knowing diagnosis by stratified analysis. A, Male patients, *P<0.05*; B, Female patients, *P<0.05*; C, the patients younger than 45, *P<0.05*; D, the patients aging from 45-55, *P<0.05*; E, the patients aging from 55-65, *P<0.05*; F, the patients aging from 65-75, *P<0.05*; G, the patients older than 75, *P<0.05*; H, the patients at stage 0-I, *P<0.05*; the patients at stage II, *P<0.05*; J, the patients at stage III, *P=0.501*; K, the patients at stage IV, *P=0.056*; L, the patients' cancer stages were unclassified, *P<0.05*; M, the patients with surgery history, *P<0.05*; N, the patients without surgery history, *P<0.05*; O, the patients diagnosed before 2006; P, the patients diagnosed between 2007-2011, *P<0.05*; Q, the patients diagnosed between 2012-2016, *P<0.05*; R, the patients reported from primary grade hospital, *P<0.05*; S, the patients reported from middle grade hospital, *P<0.05*; T, the patients reported from high grade hospital, *P<0.05*.
