# Supplementary material for: Knowledge of a cancer diagnosis is a protective factor for the survival of patients with breast cancer: a retrospective cohort study
Source: BMC Cancer. 2021 Jun 27;21:739. doi: 10.1186/s12885-021-08512-1 (PMC8237449; doi:10.1186/s12885-021-08512-1)
Supplement: Supplementary file 4 — Additional file 4. Demographic and clinical characteristics of patients with or without surgery history. [file 12885_2021_8512_MOESM4_ESM.docx]

| **Variable** | **Total number**  **(N=12327)** | **surgery history**  **(n=6697)** | **No surgery history**  **(n=5630)** | **P^*^** |
| --- | --- | --- | --- | --- |
| **Knowing status of cancer diagnosis ^a^** |  |  |  | **<0.001** |
| Did know | 9466（77.45） | 5320（79.68） | 4146（74.77） |  |
| Did not know | 2756（22.55） | 1357（20.32） | 1399（25.23） |  |
| **Sex** |  |  |  | **0.921** |
| Male | 93（0.74） | 51（0.76） | 42（0.75） |  |
| Female | 12234（99.26） | 6646（99.24） | 5588（99.25） |  |
| **Age(years)** |  |  |  | **0.018** |
| <45 | 1944（15.77） | 1039（15.51） | 905（16.07） |  |
| 45- | 3821（31.00） | 2071（30.92） | 1750（31.08） |  |
| 55- | 3488（28.30） | 2010（30.01） | 1478（26.25） |  |
| 65- | 1789（14.51） | 981（14.65） | 808（14.35） |  |
| ≥75 | 1285（10.42） | 596（8.90） | 689（12.24） |  |
| **Clinical stage** |  |  |  | **<0.001** |
| Stage 0- I | 3640（29.54） | 2083（31.10） | 1558（27.67） |  |
| Stage II | 3700（30.02） | 2201（32.86） | 1499（26.62） |  |
| Stage Ш | 1136（9.21） | 629（9.40） | 507（9.00） |  |
| Stage Ⅳ | 517（4.19） | 193（2.88） | 324（5.75） |  |
| Unclassified | 3334（27.05） | 1592（23.77） | 1742（30.94） |  |
| **Diagnostic year ^b^** |  |  |  |  |
| 1: before 2006 | 3268（26.51） | 1259（18.80） | 2009（35.68） | **<0.001** |
| 2: 2007 - 2011 | 4074（32.83） | 2420（36.14） | 1654（29.38） |  |
| 3: 2012 - 2016 | 4985（40.44） | 3018（45.06） | 1967（34.94） |  |
| **Hospital grade** |  |  |  | **<0.001** |
| Primary grade hospital | 166（1.35） | 62（0.92） | 104（1.85） |  |
| Middle grade hospital | 5088（41.28） | 2502（37.36） | 2586（45.93） |  |
| High grade hospital | 7073（57.38） | 4133（61.71） | 2940（52.22） |  |

**Additional file 4 Demographic and clinical characteristics of patients with or without surgery history, n (%)**

^*^overall comparison of surgery history by liner association chi-square in subgroups;

^a^ patients with unclear knowing status of cancer diagnosis were not included；

^b^ Diagnostic year 1 means those being diagnosed before 2006, 2 means those being diagnosed from 2007 to 2011, 3 mean those being diagnosed from 2012 to 2016.
