# Supplementary material for: Knowledge of a cancer diagnosis is a protective factor for the survival of patients with breast cancer: a retrospective cohort study
Source: BMC Cancer. 2021 Jun 27;21:739. doi: 10.1186/s12885-021-08512-1 (PMC8237449; doi:10.1186/s12885-021-08512-1)
Supplement: Supplementary file 5 — Additional file 5. Demographic and clinical characteristics of participants with unclassified clinical stage. [file 12885_2021_8512_MOESM5_ESM.docx]

| **Variable** | **Total sample**  **(N=12327)** | **Unclassified clinical stage**  **(n=3334)** |
| --- | --- | --- |
| **Knowing status of cancer diagnosis** |  |  |
| Did know | 9466(76.79) | 2139(64.16) |
| Did not know | 2756(22.36) | 1116(33.47) |
| Unclassified | 105(0.85) | 79(2.37) |
| **Sex** |  |  |
| Male | 93(0.74) | 36(1.08) |
| Female | 12234(99.26) | 3298(98.92) |
| **Average age** | 57.10±12.70 | 59.41±5.45 |
| <35 | 380(3.08) | 102(3.06) |
| 35- | 1564(12.69) | 386(11.58) |
| 45- | 3821(31.00) | 920(27.59) |
| 55- | 3488(28.30) | 853(25.58) |
| 65- | 1789(14.51) | 503(15.09) |
| ≥75 | 1285(10.42) | 571(17.13) |
| **Surgery history** |  |  |
| Yes | 6697(54.33) | 1592(47.75) |
| No | 5630(45.67) | 1742(52.25) |
| **Diagnostic year ^a^** |  |  |
| 1: before 2006 | 3268(26.51) | 1133(33.98) |
| 2: 2007 - 2011 | 4074(32.83) | 1076(32.27) |
| 3: 2012 - 2016 | 4985(40.44) | 1125(33.74) |
| **Hospital grade** |  |  |
| Primary grade hospital | 166(1.35) | 106(3.18) |
| Middle grade hospital | 5088(41.28) | 1261(37.82) |
| High grade hospital | 7073(57.38) | 1967(59.90) |

**Additional file 5 Demographic and clinical characteristics of participants with unclassified clinical stage, n(%)**

^a^ Diagnostic year 1 means those being diagnosed before 2006, 2 means those being diagnosed from 2007 to 2011, 3 mean those being diagnosed from 2012 to 2016.
